# Leisure time activities in adolescence in the presence of susceptibility genes for obesity: risk or resilience against overweight in adulthood? The HUNT study

**DOI:** 10.1186/1471-2458-12-820

**Published:** 2012-09-22

**Authors:** Koenraad Cuypers, Karin De Ridder, Kirsti Kvaløy, Margunn Skjei Knudtsen, Steinar Krokstad, Jostein Holmen, Turid Lingaas Holmen

**Affiliations:** 1 HUNT Research Center, Department of Public Health and General Practice, Faculty of Medicine, Norwegian, University of Science and Technology, Forskningsveien 2, 7600, Levanger, Norway; 2 Department of Public Health and General Practice, Faculty of Medicine, Norwegian University of Science and Technology, NTNU, Trondheim, 7491, Norway; 3 Department of Physical Medicine and Rehabilitation, Levanger Hospital, Nord-Trøndelag Health Trust, Kirkegata 2, Levanger, 7600, Norway; 4 Department of Health Promotion, Nord-Trøndelag County Council, Seilmakersgata 2, Steinkjer, 7735, Norway; 5 Psychiatric Department, Levanger Hospital, Nord-Trøndelag Health Trust, Kirkegata 2, Levanger, 7600, Norway

**Keywords:** Leisure time activities, Adolescents, Adults, Obesity, Overweight, Obesity-susceptibility loci, Genetic predisposition score

## Abstract

**Background:**

Environment, health behavior, and genetic background are important in the development of obesity. Adolescents spend substantial part of daily leisure time on cultural and social activities, but knowledge about the effects of participation in such activities on weight is limited.

**Methods:**

A number of 1450 adolescents from the Norwegian HUNT study (1995–97) were followed-up in 2006–08 as young adults. Phenotypic data on lifestyle and anthropometric measures were assessed using questionnaires and standardized clinical examinations. Genotypic information on 12 established obesity-susceptibility loci were available for analyses. Generalized estimating equations were used to examine the associations between cultural and social activities in adolescence and adiposity measures in young adulthood. In addition, interaction effects of a genetic predisposition score by leisure time activities were tested.

**Results:**

In girls, participation in *cultural* activities was negatively associated with waist circumference (WC) (B = −0.04, 95%CI: -0.08 to −0.00) and with waist-hip ratio (WHR) (B = −0.058, 95%CI: -0.11 to −0.01). However, participation in *social* activities was positively associated with WC (B = 0.040, CI: 0.00 to 0.08) in girls and with BMI (B = 0.027, CI: 0.00 to 0.05) in boys. The effect of the obesity-susceptibility genetic variants on anthropometric measures was lower in adolescents with high participation in cultural activities compared to adolescents with low participation.

**Conclusion:**

This study suggests that the effects of cultural activities on body fat are different from the effects of participation in social activities. The protective influence of cultural activities in female adolescents against overweight in adulthood and their moderating effect on obesity-susceptibility genes suggest that even cultural activities may be useful in public health strategies against obesity.

## Background

Development of obesity is influenced by a complex interplay between genetic background, environmental factors, behavior, and socio-economic status [1]. Genes are believed to be expressed where the environment allows [2]. Physical activities have previously been suggested as important leisure time activities protecting against development of obesity [3], but a substantial part of the daily leisure time in adolescence is spent on other activities, like cultural and social activities. *Cultural activities* include activities believed to require mental activities and may be performed without the company of others like reading a book, listening to or playing music or doing homework. *Social leisure time activities* imply leisure time activities performed together with friends as hanging out with friends or attend meetings or clubs.

Population based studies and human intervention studies have shown that cultural activities are associated with increased survival rate [4], better health [5] and decreased risk of dementia [6]. Recent studies on humans [7] and rodents which were placed in enriched environments where they were offered mentally exercises (i.e. toys, change in food locations, more complex housing) [8] have revealed biological and psycho-neuro-immunological pathways which may help to explain the possible health effects of these cultural activities. On the other hand, social influences may be an important cause of low compliance in physical activity interventions [9], and obesity appears to spread through social ties [10].

Adolescence may be a period when risks for chronic conditions are established through learned health behavior or emotional experiences [11]. The transition between adolescence and adulthood is a developmentally sensitive time during which adolescents may become overweight, which may last into later adulthood [12].

While the effects of physical activity have been extensively studied, the potential role of other leisure time activities in weight development from adolescents to adulthood, a period with large biological, psychological changes, has not been explored. The aim of this study was, therefore, to investigate the associations between participation in cultural and social leisure time activities in adolescence and body fat in young adulthood 11 years later. We also wanted to study whether cultural or social activities would moderate the cumulative effect of 12 obesity-susceptibility gene variants on adiposity measures at follow-up.

## Methods

### Study population

The Nord-Trøndelag Health Study, HUNT, is a large population based study, carried out in three surveys in Nord-Trøndelag County, Norway (http://www.ntnu.no/hunt) during the last 25 years: HUNT1 (1984–86), HUNT 2 (1995–97) and HUNT 3 (2006–08). The population (130 000 inhabitants) is Caucasian, homogenous, and fairly representative of Norway as a whole regarding geography, demography and occupation, but lacks large cities [13]. The average income and mean educational level is slightly lower than the national average, nevertheless the socioeconomic inequality in mortality is at the national level [14].

Young-HUNT1 was carried out in 1995–97 as the adolescent part of HUNT2. A total of 8408 (participation rate: 83%) of all students in junior and senior high schools completed a comprehensive questionnaire about lifestyle, health, and quality of life and underwent a clinical examination including anthropometric measurements. When excluding adolescents who reported to be mentally or physically disabled at baseline and who were pregnant at follow-up, 1450 people participated both in Young-HUNT 1 as adolescent (13–19 years) and in HUNT3 as young adult (24–30 years). These participants had blood samples taken in HUNT3 and were genotyped (Additional file 1: Table S1). Out of this group, 1123 participants had a baseline BMI within the normal range [15] (Additional file 1: Table S2-S3).

In both surveys, self-administered questionnaires were used to collect information on physical and mental health, somatic complaints and lifestyle. Anthropometric measures were obtained by specially trained nurses using the same standardized protocols as in Young-HUNT1. In both Young-HUNT1 (baseline) and HUNT3 (follow-up) height and weight were measured to the nearest centimetre, respectively to the nearest half kilogram with light clothes, without shoes, jacket or outdoor garments. Waist circumference (WC) was measured to the nearest centimetre, applying a non-stretchable band horizontally at the umbilical level after the participants emptied their lungs, or midway between the last rib and the iliac cristae if the latter was largest. Hip circumference was likewise measured at the thickest part of the hip [13].

### Measures at baseline: young-HUNT 1 (1995–97)

Weight status in adolescence was defined by BMI cut-offs for the appropriate age groups proposed by Cole et al. [15] Central adiposity was measured by waist circumference (WC) and waist-hip ratio (WHR).

*Cultural activities* included reading a book you liked, listening to music or playing an instrument longer than 15 min, watching TV or a video, and doing homework or school tasks longer than one hour.

*Social leisure time* activities included visiting someone you know, receiving a visit, being out for more than two hours with friends, being at a meeting or training in an organisation or a club.

In regard to cultural and social activities we assigned values from 1 to 4 for each answer-category regarding frequency (Not once (1), Once (2), 2–3 times (3), 4 times or more (4) in the last 7 days) and constructed a variable by adding the scores for cultural and social activities separately (range 4–16). Both results of earlier research [10,16,17] and statistical analyses [18] support our choice of the components in the definition of cultural and social activities. In the multivariable analyses this variable was assumed to be continuous. In the stratified analyses we dichotomised and converted the cultural variable into high culturally active corresponding to two or more cultural activities a day (1). High participation (1) in social activities was defined as corresponding to doing more than one social activity a day.

Self-reported pubertal development status was assessed based on the Pubertal Development Scale (PDS) [19].

### Measures at follow-up, HUNT 3 (2006–08)

According to the WHO recommendations overweight was defined as BMI ≥ 25 kg/m^2^[20]. Central overweight was defined as WC ≥ 80 cm for females and WC ≥ 94 cm for men [21]. Body fat distribution was measured by WHR. Overweight was defined as WHR > 0.80 in women and > 0.90 in men [22].

Z-scores were calculated to be able to adjust for age and sex and to pool all body fat estimates data of the adolescents and young adults, accounting for the natural development of the body measures over age in a longitudinal life perspective. The z-scores of the respective adiposity measures (at baseline sex-and-age specific and at follow-up sex-specific) were used as continuous variables.

The physical activity level was assessed by self-report as described previously for adolescents and adults, and four categories of physical activity change over time were defined (stable active, stable inactive, from inactivity to activity and from activity to inactivity [23]. We chose to use this “pattern”-physical activity variable because there is indication that changes in physical activity may be more predictive in concordance to Kettaneh et al. [24].

Socio-economic status (SES) was measured in HUNT 3 (2006–08) reclassifying the Norwegian occupation classification (STYRK) into an approximation to the Erikson Goldthorpe Portocarero social class scheme [14].

### Genotypes

In all, 12 SNPs representing 12 susceptibility loci associated with body fat estimates in GWA studies [25-29] and recently replicated in adolescent [30-33] were genotyped: rs6265 in *BDNF*, rs545854 near *MRSA*, rs571312 near *MC4R*, rs987237 near *TFAP2B*, rs1121980 in *FTO*, rs281575*2* near *NEGR1*, rs6548238 near *TMEM18*, rs10195252, near *GRB14*, rs10838738 in *MTCH2*, rs10938397 near *GNPDA2*, rs7566605 near *INSIG2*, and rs11084753 near *KCTD15*. Genotyping was carried out by Centre for Integrative Genetics (CIGENE), Norwegian University of Life Sciences, Norway. All variants passed quality-control criteria with a call-rate ≥ 95% and were in Hardy-Weinberg equilibrium (p > 0.05), assessed by the genetic analysis program PLINK (Additional file 1: Table S1).

Because a genetic predisposition score GPS might reflect larger effects than each single variant separately [34] a GPS across the 12 SNPs was calculated by summing the BMI-increasing alleles [35] defined by the results reported by recent GWA studies [25-31,36]. Individuals with missing genotypes for more than three SNPs were excluded (0.6% of the sample).

For the purpose of calculating the genetic predisposition score in individuals with missing genotypes for three SNPs or less, missing genotype data were replaced with the average allele count of the respective SNP. The GPS was normally distributed (n = 1450, mean: 11.4, SD: 2.2) and inserted in the analyses as a continuous variable.

### Ethics

Participation in the HUNT and the Young HUNT study was voluntary and approved by the Norwegian Data Inspectorate, The Directorate of Health, and by the Regional Committee for Medical and Health Research Ethics. All participants and the guardians of adolescents younger than 16 years signed an informed consent.

### Statistics

Social or cultural activities in adolescence were divided into quartiles where first quartile indicated lowest participation rate and fourth quartile highest participation rate. In each group the prevalence of the weight status (normal weight and overweight) in young adulthood was recorded.

Separate Generalized Estimating Equation (GEE) type linear models were employed to study the independent relationships between participation in cultural activities and social activities at baseline and z-scores of BMI, WC, and WHR respectively at follow-up. Because the effect of environmental influences seems to be larger in individuals with phenotypic extremes during adolescence [37] and BMI at baseline may affect both participation in leisure time activities and tracking of BMI into adulthood, we chose to present the analyses including only the 1123 adolescents with normal weight at baseline [38].

GEE adjusts for the correlation between repeated observations taken in the same individual. In all these analyses, an exchangeable correlation structure was assumed. We adjusted for pubertal development at baseline, physical activity change, and SES at follow-up simultaneously.

We chose the components included in “cultural activity” and “social activity” based on knowledge of adolescent’s preferred activities at the time from previous literature [16,18] and from the choices as found in a study of the first author including adolescents from the same area [17]. Also, all the components chosen for the “cultural activity”-index were activities believed to require mental activity. TV-viewing is an important leisure time activity in adolescents and was included. However, the direction of the association between TV/video-viewing and obesity is equivocal and may be biased by snacking and psycho-social problems [39-41]. We chose the components included in “social activity”-index because we assumed the adolescents to socially meet during these activities. In order to be sure of directional consistency in our data each component in both the cultural and social index was tested in separate models all showing the same direction in each index.

In additional GEE-models we investigated interaction between participation in social and cultural activities by introducing the interaction terms (cultural activities*social activities) in addition to the main effect of the leisure time activity to see whether participation in one kind of leisure time activity modified the effect of the other. Gender specific models were calculated due to statistically significant interactions between gender and both leisure time activities.

With linear regressions models, we studied the association between the GPS and z-scores for BMI and WC at follow-up respectively. The interaction effect between the respective leisure time activities (cultural and social) and GPS on the association with z-scores BMI and z-scores WC at follow-up was assessed. We checked interaction by introducing cross-product terms (participation in social activities*GPS and participation in cultural activities*GPS) in addition to the main effect of the respective leisure time activity and GPS. Additionally, we also investigated the relationship between the z-scores of BMI and WC and the GPS in those with high and in those with low participation in social and cultural activities. Interaction between GPS and gender was tested by including the cross-product term (GPS*gender) in the model, in addition to the main effect of GPS and gender on the adiposity traits.

Statistical analyses were performed using IBM SPSS Statistics, version 19. A p-value at or below 0.05 and a 95% confidence interval denoted statistical significance. In regard to the interaction terms we used the p < 0.10 level, because an interaction term is a multiplication of two variables which both include measurement error, resulting in a multiplicative error and subsequent high standard error.

## Results

In the analyses of associations between participation in cultural or social activities and adiposity measures at follow-up, a total of 775 girls and 675 boys were included. More girls than boys were highly engaged in *cultural* activities, while participation in *social* activities was equally distributed between the genders.

Descriptive analyses (Table 1) showed an inverse relationship between frequency of participation in *cultural* activities in adolescence and overweight in young adulthood in both normal weight girls and boys. P for trend in the four quartiles was beyond the 0.01 level for all three adiposity measures in young adulthood. In contrast, there was a positive association between participation in *social* activities in adolescence and overweight in young adulthood; however, the P for trend did not reach statistical significance.

**Table 1:** Mean adiposity measures and standard deviation at young adulthood in the four quartiles of participation in leisure time activities in adolescence from 1 (lowest) to four (highest)

	**Cultural activities**						**Social activities**					
	**BMI**		**WC**		**WHR**		**BMI**		**WC**		**WHR**	
1	25.0	3.2	88.6	9.3	0.869	0.056	24.6	3.3	85.8	10.9	0.849	0.074
2	25.1	3.5	87.7	10.6	0.860	0.072	24.6	3.4	85.7	10.6	0.845	0.066
3	24.9	3.6	87.2	11.2	0.854	0.070	24.9	3.5	86.7	10.6	0.853	0.069
4	24.3	3.5	84.3	10.9	0.834	0.069	25.0	3.6	87.1	10.8	0.853	0.069
P	0.006		<0.001		<0.001		0.10		0.06		0.23	

Girls n = 603; Boys n = 520. Excluded: overweight and underweight, pregnant, disabled physically or psychologically in daily activities.

P: p for trend. Cultural leisure time activities includes reading a book you like, listening to music or played an instrument longer than 15 min, watching TV or a video, and doing home work or school tasks longer than an hour. Social leisure time activities included visiting some one you know, receiving a visitor, being out for more than two hours with friends, and being at a meeting or training in an organization or a club. We assigned values from 1 to four for each answer-category regarding frequency Note once (1), Once (2), 2–3 times, (3), 4 times or more (4) in the last seven days and made a continuous variable by adding the scores for each activity (range 4–16). Then 4 quartiles were constructed from low to high participation.

### Participation in cultural activities or social activities and adiposity measures in adulthood

Including all girls and boys (n = 1450), an inverse association between participation in *cultural* activities at baseline and z-scores of WC and WHR in adulthood was found in girls, but not in boys. In boys, participation in social activities was significantly positively associated with z-scores of BMI and WC and near-significant with z-scores of WHR in adulthood (Additional file 1: Table S4 and Table S5).

In the same models including only those with normal weight (n = 1123) at baseline, the analyses pictured similar results, but now the associations were statistically significant (Table 2) in relation to the cultural activities. In an additional model (Additional file 1: Table S6) we excluded “watching TV or a video” as a component from the index cultural activities. In the model 2 adjusted for relevant confounders the effect estimates moved slightly toward zero from -.040 (−.08 to 0) to -.034 (−.07 to 0) and from -.058 (−.11 to -.01) to – .046 (−.08 to -.01) for the z-scores of WC and WHR respectively.

**Table 2:** Associations between the index for cultural activities at baseline and the z-scores of body mass index (BMI), waist circumference (WC), and waist-hip ratio (WHR) eleven years later as adults

			**Girls**				**Boys**	
	**B**	**SE**	**P**	**CI (95%)**	**B**	**SE**	**P**	**CI (95%)**
BMI								
Model 1	-.015	.013	.25	-.04 to .01	-.015	.013	.25	-.04 to .01
Model 2	-.011	.019	.58	-.05 to .03	.007	.020	.74	-.03 to .05
WC								
Model 1	-.028	.015	.05	-.06 to 0	-.012	.014	.36	-.04 to .01
Model 2	-.040	.021	.05	-.08 to 0	.001	.020	.97	-.04 to .04
WHR								
Model 1	-.048	.016	.002	-.08 to -.02	-.020	.017	.23	-.05 to.01
Model 2	-.058	.025	.02	-.11 to -.01	.003	.023	.88	-.05 to .04

Girls n = 603; Boys n = 520. Excluded: overweight and underweight, pregnant, disabled physically or psychologically in daily activities.

Model 1 Unadjusted effect of exposure on the outcome measure at follow-up

Model 2. Adjusted pubertal development at baseline, physical activity change from adolescence to adulthood, and socio-economic status at follow-up.

Cultural activities: reading a book you liked, listening to music or played an instrument longer than 15 min, watching TV or a video, and doing homework or school tasks longer than an hour. We assigned values from 1 to 4 for each answer-category regarding frequency (Not once (1), Once (2), 2–3 times (3), 4 times or more (4) in the last 7 days) and made a continuous variable by adding the scores for each activity (range 4–16).

Employed GEE linear type model. (N: 1123 normal-weight adolescents).

In relation to the social activities the confidence intervals became broader (Table 3). Participation in *social* activities in girls was significantly positively associated with z-scores of WC in adulthood (Table 3). In boys the relationship between participation in social activities and BMI z-scores was still significant and near significant with WC. In addition, the associations for all activities separately toward the outcome showed the same direction in linear regression models as for the indices of cultural and social activities.

**Table 3:** Associations between the index for social activities at baseline and the z-scores of body mass index (BMI), waist circumference (WC), and waist-hip ratio (WHR) eleven years later as adults

			**Girls**				**Boys**	
	**B**	**SE**	**P**	**CI (95%)**	**B**	**SE**	**P**	**CI (95%)**
BMI								
Model 1	.016	.011	.14	-.01 to .04	.009	.009	.37	-.01 to .03
Model 2	.022	.017	.20	-.01 to .06	.027	.013	.05	.0 to .05
WC								
Model 1	.019	.012	.11	.0 to .04	.008	.011	.46	-.01 to .03
Model 2	.040	.019	.03	.0 to .08	.029	.015	.06	.0 to .06
WHR								
Model 1	.015	.013	.24	-.01 to .04	.020	.012	.12	-.01 to .04
Model 2	.027	.022	.21	-.02 to .07	.024	.018	.18	-.01 to .06

Girls n: 603; Boys n: 520. Excluded: overweight and underweight, pregnant, disabled physically or psychologically in daily activities.

Model 1 Unadjusted effect of exposure on the outcome measure at follow-up.

Model 2. Adjusted in for pubertal development at baseline, physical activity change, socio-economic status at follow-up.

Social activities: visiting some one you know, receiving a visitor, being out for more than two hours with friends, being at a meeting or training in an organization or a club.

We assigned values from 1 to 4 for each answer-category regarding frequency (Not once (1), Once (2), 2–3 times (3), 4 times or more (4) in the last 7 days) and made a continuous variable by adding the scores for each activity (range 4–16).

Employed GEE linear type model. (N: 1123 normal-weight adolescents).

We have chosen to show the results for the models including only normal weight adolescents at baseline in the paper (Tables 2 and 3) and have attached data for the whole population in the Additional file 1: Tables S4 and S5.

### Interaction between cultural and social activities

No significant correlation (r =0.05, p = 0.12 and r = 0.03, p = 0.39 in girls and boys respectively) and no interaction (p = 0.60) was noted between participation in social and cultural activities, indicating that cultural and social leisure time activities were independent concepts in their relationship to the adiposity measures.

### Moderation of the effect of susceptibility genes for obesity on adiposity measures by leisure time activities

The GPS was significantly associated with both continuous traits z-scores of BMI (0.03 SD/allele, 95% CI: 0.01 to 0.05, p = 0.008) and z-scores of WC (0.03 SD/allele, 95% CI: 0.06 to 0.05, p = 0.012) at follow-up.

**Figure 1:**
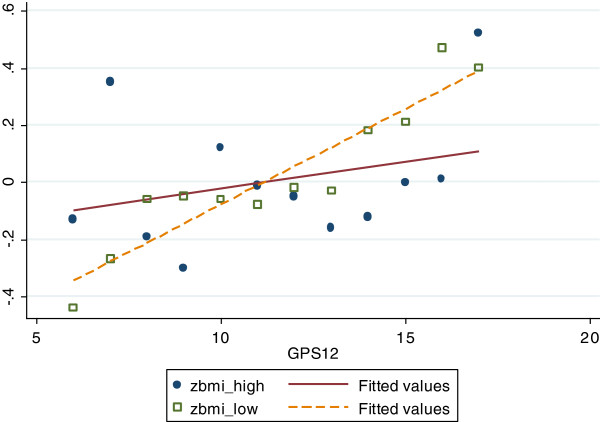
Z-scores of BMI with different genetic predisposition scores in low versus high culturally active individuals.

**Figure 2:**
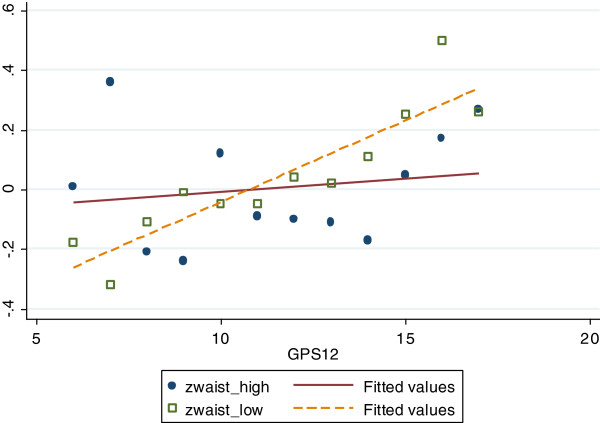
Z-scores of Waist circumference with different genetic predisposition scores in low versus highly culturally active individuals.

Also, in those with low cultural activity, the GPS had a substantial and significant (p for interaction = 0.06) higher effect size (SD/allele: 0.050 (95% CI: 0.02 to 0.08) versus 0.005 (95% CI: -0.03 to 0.04)) (Figure 1) on BMI 11 years later compared to the group with high culturally activity. The same trends were seen for WC (Figure 2). We found no significant interactions between neither gender nor *social* activity participation and the GPS in association with adiposity measures in adulthood.

## Discussion

Our data showed that adolescent girls who participated in *cultural* activities had less central adiposity eleven years later compared to girls who did not participate in such activities. However, participation in *social* leisure time activities predicted *increased* central adiposity in both girls and boys. In adolescent boys particularly, participation in social leisure time activities predicted higher overall adiposity at follow-up. Additionally, highly culturally active adolescents seemed to be better protected against the effect of obesity-susceptibility genes when measured in young adulthood.

Strengths of the present study are a general population sample and the 11 year follow-up of adolescents into adulthood (Additional file 1: Tables S2 and S3). The great mobility in this age group and consequently loss of follow-up participants was a challenge. However, comparison of baseline data between participants and non-participants in the follow-up showed no essential differences, suggesting no systematic selection bias (data not shown). Socio-economy might have an important influence on participation in cultural and social activities, though adjusting for the socio-economic status in adulthood did not change the results.

Regression to the mean (RTM) in the two subsequent measurements may occur. In this way, the RTM-effect may have decreased the observed association. Although, a certain possible measurement error was likely to behave in the opposite direction, the adiposity measures were very homogenous in the study sample and were not expected to affect the associations substantially.

We repeated the analyses including only the 1123 adolescents with normal weight at baseline in the analyses, because the effect of environmental influences are suggested to be larger in individuals with phenotypic extremes during adolescence [37] and might bias our results. Additionally, overweight in adolescents tends to track into adulthood [42] and it is more likely that environmental effects might be easier detectable in normal weight adolescents without onset of obesity. The results might indicate that the favourable effect of cultural participation in adolescence is more preventive against overweight and less a remedy for becoming normal weight again.

A possible limitation might be that we did not include physical activity as a component in the index of social activity. We were mostly interested in the “social” part of the social activities, although we cannot exclude some overlap with physical activity in our definition of social activities. Therefore, we adjusted for the level of physical activity.

### The effect of cultural participation

There might be several explanations for the inverse effect of participation in cultural activities on adiposity measures. Firstly, participation in cultural activities is found to be associated with an overall regular lifestyle and healthy behaviour [43]. Secondly, relaxing leisure time activities like listening to music, reading and TV watching seem to be strong predictors of coping with stress, as suggested by Iwasaki et al. [44] and thereby counteract stress-induced central obesity. Thirdly, Lajunen et al. [16] argues that adolescents cannot eat while playing music, keeping the energy input and output in balance.

Girls were obviously more engaged in cultural activities compared to boys, and it seemed that only in girls the participation in cultural activities was protective against overweight. This gender difference may be due to factors like different food choices, body satisfaction, and physical activity , as well as differences in participation in cultural activities [45].

Our findings might possibly be related to some biological pathways, demonstrated in animal experiments. Rodents who were exposed to enriched environments where they were offered exercises both mentally (i.e. toys, change in food locations, more complex housing) and physically tended to switch from a white fat phenotype to a brown fat phenotype, more likely to burn fat depots [46]. Furthermore, diet induced obesity has been shown to be prevented through the genetic and environmental activation of hypothalamic-adipocyte axis [8]. These effects were specifically noticed in rodents exposed to an enriched environment compared to rodents exposed only to physical exercise, like running. Histological studies show that these mechanisms may also be relevant in humans [47].

Cultural activities, as defined in this study, were sitting activities. There is no unanimous conclusion on the negative effects of the different sedentary activities per se on development of overweight [43]. Although there is evidence that TV viewing is a risk factor for development of obesity, being more important in children than adolescents [48], the pathways of the association are unclear [39-41]. We have, therefore, repeated the analyses without the component “Watching TV or a video” as a part of the index of cultural activities (Additional file 1: Table S6). The exclusion of “Watching TV or a video” from the cultural activity index did not change the results substantially. Specifically, excluding the component TV viewing from the index of cultural activities weakened the effect estimates slightly, but the confidence intervals remained nearly unchanged. “Watching TV or a video” was individually also inversely associated with central body fat estimates in girls in discordance with other studies [49,50]. TV viewing may in our population counteract stress in concordance to Iwasaki et al. [44]. Another explanation may be that TV viewing seemed not to displace physical activity in our population. Biddle et al. has stated that TV viewing may be reflective of other unhealthy behaviours [51], but this does not seem to be the case for the girls in our study. Also, the relationship between TV viewing and obesity has been suggested to be influenced by other confounders like snacking, psycho-pathologies [39], socio-economic status [51] and family structure [48] which may not be applicable to the girls in our population. Finally, Norwegian adolescents seemed to consume less TV during dinner than peers in other European countries [49].

### The effect of social activities

Consistent with the findings of Christakis et al. [10], but in discordance with the study of Lajunen et al. [16], our data suggest a positive association between adolescent participation in *social* activities and overweight in young adulthood. The social activities as used in the study of Lajunen comprised not solely social activities. Like Barabasi et al. [52], we might suggest that the social network may be a factor in the spread of obesity through social ties. Some studies [6,53] proposed psychosocial pathways as one possible mechanism in the beneficial effects of social participation on psychological health in adults. Others [4], on the contrary, found that the relative risk of mortality increased equal to the extent of the social network. Likewise, our data indicate negative influence on health measures, i.e. adiposity measures. One can imagine that in adolescents, a psychological susceptible period with diverse psychosocial influences and with peers as important reference persons, it might be difficult to sustain a good self-concept. Adolescents without good self-concept might change easily their lifestyle to the unhealthier one of their friends.

### Modification of obesity-susceptibility genetic markers

There is growing evidence that the 32 loci robustly associated in large-scale meta-analyses of GWAS with BMI in adults [25-27,29-31,36], also affect BMI in childhood and adolescence [31,32,54,55]. Heritability of BMI is estimated to vary from 0.58 among 11 year-old girls and boys to 0.83 and 0.74 respectively in 17 year-old boys and girls. Also, the genetic contribution to BMI seems to be strong during adolescence [56]. Though, the known combined genetic effect on BMI is modest, estimated to only 2–3 % of the genetic variation in BMI.

In concordance to other studies [57,58] we preferred to use a genetic predisposition score rather than a single locus because a genetic predisposition score explains also a greater genetic variance. This approach may be preferable when demonstrating an interaction between genetic susceptibility and a life style factor. Additionally, multiple allele scores increase statistical power and may represent a more generalizable influence of genetic obesity susceptibility compared with analyses of individual variants.

For estimating the cumulative effect of the SNPs combined we calculated a genetic predisposition score (GPS) by summing the BMI-increasing alleles across the 12 SNPs [35]. We did not weight the risk alleles on basis of their individual effect sizes because no well-accepted effect sizes were available for each of the SNPs. It is previously demonstrated that weighting of risk alleles may have limited effect [59].

Lajunen et al. [56] also indicated that common environmental factors had an effect in early adolescence. According to Haberstick et al. [60] the role of environment compared to genetic influence may be more profound in girls than in boys.

Our data revealed a marked divergence between girls and boys in the distribution of body fat. Participation in social activities was in girls more related to central adiposity and in boys to overall adiposity. This is in congruence with other studies [5,45]. The divergence between girls and boys may be due to biology which is expressed in differences in distribution, storage and metabolic processing of body fat. Physical activity in contrary to cultural activities seems not to attenuate the effect of obesity-susceptibility genetic variants on adiposity estimates in adolescents [61].

Eventually, environmental influences like psychosocial stress may override genetically driven factors [62] and increase particularly the central adipose measures in girls, who are more susceptible for stress-related pathology [63]. Another reason could be that the effects of genetic influences could be sex-limited due to different neuro-endrocrine functioning and fat metabolism [64]. As a consequence, an important question is whether this difference in fat distribution might be an indication of greater health risk in girls [64].

Further, from previous studies we know that the *MC4R* has a role in the food intake, thermogenesis and loco motor activity [65]. The well-known *FTO* has possible a role in the HPA-axis and influences body composition through energy expenditure [31,66,67]. Thus, a pathway that affects body composition through the central or distal nervous system might be possible. This pathway we have hypothesized also to be important for the effect of leisure time activity.

## Conclusion

Both cultural and social leisure time activities in adolescence seem to have an effect on weight development into adulthood, but in opposite directions. In girls, participation in cultural activities seemed to protect against excessive weight gain, while participation in social activities may increase overweight in both gender. This study is the first, to our knowledge, to indicate that the cumulative effect of obesity-susceptibility genes for obesity in girls and boys may be attenuated by participation in cultural activities.

## Competing interests

The authors declare no competing interests or any financial interests.

## Authors' contributions

All authors have participated in the development and writing of this study. All authors read and approved the final manuscript.

## Pre-publication history

The pre-publication history for this paper can be accessed here:

http://www.biomedcentral.com/1471-2458/12/820/prepub

## Supplementary Material

Additional file 1 Table S1.Genotype information and quality control statistics for the 12 obesity-susceptibility SNP. **Table S2** Characteristics of baseline adolescent population who met the inclusion criteria and participated in the follow-up study as adults. Prevalence in numbers or percentages (%), distribution in mean and standard deviation (SD). **Table S3** Characteristics of follow-up population who met the inclusion criteria and participated in the baseline study as adolescents. Prevalences are expressed in percentages (%), distribution in mean and standard deviation m (SD). **Table S4**. Associations between the index for cultural activities at baseline and the z-scores of body mass index (BMI), waist circumference (WC) and waist hip ratio (WHR) eleven years later as adults. Employed GEE linear type model. (N: all 1450 adolescents). **Table S5**. Associations between the index for social activities at baseline and the z-scores of body mass index (BMI), waist circumference (WC) and waist hip ratio (WHR) eleven years later as adults. Employed GEE linear type model. (N: all 1450 adolescents). **Table S6**. Associations between the index for cultural activities (without TV-viewing) at baseline and the z-scores of body mass index (BMI), waist circumference (WC), and waist-hip ratio (WHR) eleven years later as adults. Employed GEE linear type model. (N: 1123 normal-weight adolescents). (DOCX 33 kb) Click here for file

## References

[B1] Graff M North KE Monda KL Lange EM Lange LA Guo G Gordon-Larsen P The combined influence of genetic factors and sedentary activity on body mass changes from adolescence to young adulthood: the national longitudinal adolescent health study Diabetes Metab Res Rev 2011 27 1 63 69 10.1002/dmrr.1147 21218509 PMC3040976

[B2] Ogden CL Yanovski SZ Carroll MD Flegal KM The epidemiology of obesity Gastroenterology 2007 132 6 2087 2102 10.1053/j.gastro.2007.03.052 17498505

[B3] Ekelund U Brage S Froberg K Harro M Anderssen SA Sardinha LB Riddoch C Andersen LB TV viewing and physical activity are independently associated with metabolic risk in children: the European Youth Heart Study PLoS Med 2006 3 12 e488 10.1371/journal.pmed.0030488 17194189 PMC1705825

[B4] Bygren LO Konlaan BB Johansson SE Attendance at cultural events, reading books or periodicals, and making music or singing in a choir as determinants for survival: Swedish interview survey of living conditions BMJ 1996 313 7072 1577 1580 10.1136/bmj.313.7072.1577 8990990 PMC2359094

[B5] Cuypers KF Krokstad S Holmen LT Knudtsen SM Bygren LO Holmen J Patterns of receptive and creative cultural activities and their association with perceived health, anxiety, depression, and satisfaction with life among adults: The HUNT-study, Norway J Epidemiol Commu Heal 2012 66 8 698 703 Epub 2011 May 23 10.1136/jech.2010.113571 21609946

[B6] Wang HX Karp A Winblad B Fratiglioni L Late-life engagement in social and leisure activities is associated with a decreased risk of dementia: a longitudinal study from the kungsholmen project Am J Epidemiol 2002 155 12 1081 1087 10.1093/aje/155.12.1081 12048221

[B7] Theorell T von Scheele B Grape C Lingham J Lindblad F Sodergren A Biological effects of performing (singing) and listening to music J Psychosom Res 2006 61 3 390 390

[B8] Cao L Choi EY Liu X Martin A Wang C Xu X During MJ White to brown fat phenotypic switch induced by genetic and environmental activation of a hypothalamic-adipocyte axis Cell Metab 2011 14 3 324 338 10.1016/j.cmet.2011.06.020 21907139 PMC3172615

[B9] Loos RJ Rankinen T Tremblay A Perusse L Chagnon Y Bouchard C Melanocortin-4 receptor gene and physical activity in the Quebec family study Int J Obes (Lond) 2005 29 4 420 428 10.1038/sj.ijo.0802869 15597110

[B10] Christakis NA Fowler JH The spread of obesity in a large social network over 32 years The New England J Med 2007 357 4 370 379 10.1056/NEJMsa066082 17652652

[B11] Byrne DG Davenport SC Mazanov J Profiles of adolescent stress: the development of the adolescent stress questionnaire (ASQ) J Adolescence 2007 30 3 393 416 10.1016/j.adolescence.2006.04.004 16750846

[B12] Anderson PM Butcher KE Childhood obesity: trends and potential causes Future Child 2006 16 1 19 45 10.1353/foc.2006.0001 16532657

[B13] Holmen J MK, Krüger Ø: **The Nord-Trøndelag Health study 1995–97 (HUNT2). Objectives, contents, methods and participation** Norsk Epidemiologi 2003 13 19 32

[B14] Krokstad S Westin S Health inequalities by socioeconomic status among men in the Nord-Trondelag health study, Norway Scandinavian J Publ health 2002 30 2 113 124 10.1080/14034940210133753 12028860

[B15] Cole TJ Bellizzi MC Flegal KM Dietz WH Establishing a standard definition for child overweight and obesity worldwide: international survey BMJ 2000 320 7244 1240 1243 10.1136/bmj.320.7244.1240 10797032 PMC27365

[B16] Lajunen HR Keski-Rahkonen A Pulkkinen L Rose RJ Rissanen A Kaprio J Leisure activity patterns and their associations with overweight: a prospective study among adolescents J Adolescence 2009 32 5 1089 1103 10.1016/j.adolescence.2009.03.006 PMC2735596 19345989

[B17] Cuypers KF Gundersen KT The relations between gender, age, BMI and physically and passive cultural attendance Book of Abstracts 13Th Annual Congress of the European College of Sport Science 2008 Estoril- Portugal 111

[B18] Myklestad I Roysamb E Tambs K Risk and protective factors for psychological distress among adolescents: a family study in the Nord-Trondelag health study Soc Psychiat Psychiat Epidemiol 2012 47 5 771 782 10.1007/s00127-011-0380-x 21499806

[B19] Bratberg GH Nilsen TI Holmen TL Vatten LJ Early sexual maturation, central adiposity and subsequent overweight in late adolescence. a four-year follow-up of 1605 adolescent Norwegian boys and girls: the Young HUNT study BMC Publ Health 2007 7 54 10.1186/1471-2458-7-54 PMC1855319 17430580

[B20] WHO Consultation on Obesity. Obesity: preventing and managing the global epidemic. Report of a WHO Consultation World Health Organ Tech Rep Ser 2000 894 i xii 1-253 11234459

[B21] Han TS van Leer EM Seidell JC Lean ME Waist circumference action levels in the identification of cardiovascular risk factors: prevalence study in a random sample BMJ 1995 311 7017 1401 1405 10.1136/bmj.311.7017.1401 8520275 PMC2544423

[B22] Deshmukh A Maliye C Gupta S Bharambe M Dongre A Kaur S Garg B Does Waist-Hip ratio matter?- A study in rural india Regional Health Forum 2005 9 28 35

[B23] Rangul V Holmen TL Bauman A Bratberg GH Kurtze N Midthjell K Factors predicting changes in physical activity through adolescence: the young-HUNT Study, Norway J Adolescent Health 2011 48 6 616 624 10.1016/j.jadohealth.2010.09.013 21575823

[B24] Kettaneh A Oppert JM Heude B Deschamps V Borys JM Lommez A Ducimetiere P Charles MA Changes in physical activity explain paradoxical relationship between baseline physical activity and adiposity changes in adolescent girls: the FLVS II study Int J Obes (Lond) 2005 29 6 586 593 10.1038/sj.ijo.0802992 15889117 PMC2043091

[B25] Speliotes EK Willer CJ Berndt SI Monda KL Thorleifsson G Jackson AU Allen HL Lindgren CM Luan J Magi R Association analyses of 249,796 individuals reveal 18 new loci associated with body mass index Nat Genet 2010 42 11 937 948 10.1038/ng.686 20935630 PMC3014648

[B26] Thorleifsson G Walters GB Gudbjartsson DF Steinthorsdottir V Sulem P Helgadottir A Styrkarsdottir U Gretarsdottir S Thorlacius S Jonsdottir I Genome-wide association yields new sequence variants at seven loci that associate with measures of obesity Nat Gen 2009 41 1 18 24 10.1038/ng.274 19079260

[B27] Lindgren CM Heid IM Randall JC Lamina C Steinthorsdottir V Qi L Speliotes EK Thorleifsson G Willer CJ Herrera BM Genome-wide association scan meta-analysis identifies three Loci influencing adiposity and fat distribution PLoS Genet 2009 5 6 e1000508 10.1371/journal.pgen.1000508 19557161 PMC2695778

[B28] Heid IM Jackson AU Randall JC Winkler TW Qi L Steinthorsdottir V Thorleifsson G Zillikens MC Speliotes EK Magi R Meta-analysis identifies 13 new loci associated with waist-hip ratio and reveals sexual dimorphism in the genetic basis of fat distribution Nat Genet 2010 42 11 949 960 10.1038/ng.685 20935629 PMC3000924

[B29] Heard-Costa NL Zillikens MC Monda KL Johansson A Harris TB Fu M Haritunians T Feitosa MF Aspelund T Eiriksdottir G NRXN3 is a novel locus for waist circumference: a genome-wide association study from the CHARGE Consortium PLoS Genet 2009 5 6 e1000539 10.1371/journal.pgen.1000539 19557197 PMC2695005

[B30] Frayling TM Timpson NJ Weedon MN Zeggini E Freathy RM Lindgren CM Perry JR Elliott KS Lango H Rayner NW A common variant in the FTO gene is associated with body mass index and predisposes to childhood and adult obesity Science 2007 316 5826 889 894 10.1126/science.1141634 17434869 PMC2646098

[B31] Willer CJ Speliotes EK Loos RJ Li S Lindgren CM Heid IM Berndt SI Elliott AL Jackson AU Lamina C Six new loci associated with body mass index highlight a neuronal influence on body weight regulation Nat Gen 2009 41 1 25 34 10.1038/ng.287 PMC2695662 19079261

[B32] Zhao J Bradfield JP Li M Wang K Zhang H Kim CE Annaiah K Glessner JT Thomas K Garris M The role of obesity-associated loci identified in genome-wide association studies in the determination of pediatric BMI Obesity (Silver Spring) 2009 17 12 2254 2257 10.1038/oby.2009.159 19478790 PMC2860782

[B33] den Hoed M Ekelund U Brage S Grontved A Zhao JH Sharp SJ Ong KK Wareham NJ Loos RJ Genetic susceptibility to obesity and related traits in childhood and adolescence: influence of loci identified by genome-wide association studies Diabetes 2010 59 11 2980 2988 10.2337/db10-0370 20724581 PMC2963559

[B34] Sieberts SK Schadt EE Moving toward a system genetics view of disease Mammalian genome : official journal of the International Mammalian Genome Society 2007 18 6–7 389 401 17653589 10.1007/s00335-007-9040-6 PMC1998874

[B35] Li S Zhao JH Luan J Luben RN Rodwell SA Khaw KT Ong KK Wareham NJ Loos RJ Cumulative effects and predictive value of common obesity-susceptibility variants identified by genome-wide association studies Am J Clin Nutr 2010 91 1 184 190 10.3945/ajcn.2009.28403 19812171

[B36] Loos RJ Lindgren CM Li S Wheeler E Zhao JH Prokopenko I Inouye M Freathy RM Attwood AP Beckmann JS Common variants near MC4R are associated with fat mass, weight and risk of obesity Nat Gen 2008 40 6 768 775 10.1038/ng.140 PMC2669167 18454148

[B37] Nelson MC Gordon-Larsen P North KE Adair LS Body mass index gain, fast food, and physical activity: effects of shared environments over time Obesity (Silver Spring) 2006 14 4 701 709 10.1038/oby.2006.80 16741273

[B38] Wardle J Carnell S Haworth CM Plomin R Evidence for a strong genetic influence on childhood adiposity despite the force of the obesogenic environment Am J Clin Nutr 2008 87 2 398 404 18258631 10.1093/ajcn/87.2.398

[B39] Heim J Brandtzæg PB Kaare BH Endestad T Torgerson L Children's usage og media technologies and psychosocial factors New Media and Soc 2007 9 425 454 10.1177/1461444807076971

[B40] Marshall SJ Biddle SJ Gorely T Cameron N Murdey I Relationships between media use, body fatness and physical activity in children and youth: a meta-analysis International journal of obesity and related metabolic disorders : journal of the International Association for the Study of Obesity 2004 28 10 1238 1246 10.1038/sj.ijo.0802706 15314635

[B41] Biddle SJ Gorely T Marshall SJ Murdey I Cameron N Physical activity and sedentary behaviours in youth: issues and controversies J R Soc Promot Health 2004 124 1 29 33 10.1177/146642400312400110 14971190

[B42] Singh AS Mulder C Twisk JW van Mechelen W Chinapaw MJ Tracking of childhood overweight into adulthood: a systematic review of the literature Obesity reviews: an official journal of the International Association for the Study of Obesity 2008 9 5 474 488 10.1111/j.1467-789X.2008.00475.x 18331423

[B43] Utter J Neumark-Sztainer D Jeffery R Story M Couch potatoes or french fries: are sedentary behaviors associated with body mass index, physical activity, and dietary behaviors among adolescents? J Am Dietetic Assoc 2003 103 10 1298 1305 10.1016/S0002-8223(03)01079-4 14520247

[B44] Iwasaki Y Counteracting stress through leisure coping: a prospective health study Psychol Health Med 2006 11 2 209 220 10.1080/13548500500155941 17129909

[B45] Sweeting HN Gendered dimensions of obesity in childhood and adolescence Nutr J 2008 7 1 10.1186/1475-2891-7-1 18194542 PMC2265740

[B46] van Praag H Kempermann G Gage FH Neural consequences of environmental enrichment Nat Rev Neurosci 2000 1 3 191 198 11257907 10.1038/35044558

[B47] Cinti S De Matteis R Zingaretti MC Murano I Vitali A Frontini A Giannulis I Barbatelli G Marcucci F Bordicchia M In vivo physiological transdifferentiation of adult adipose cells Stem Cells 2009 27 11 2761 2768 10.1002/stem.197 19688834

[B48] Rey-Lopez JP Vicente-Rodriguez G Biosca M Moreno LA Sedentary behaviour and obesity development in children and adolescents Nutr Metabol Cardiovasc Dis: NMCD 2008 18 3 242 251 10.1016/j.numecd.2007.07.008 18083016

[B49] te Velde SJ De Bourdeaudhuij I Thorsdottir I Rasmussen M Hagstromer M Klepp KI Brug J Patterns in sedentary and exercise behaviors and associations with overweight in 9–14-year-old boys and girls–a cross-sectional study BMC Public Health 2007 7 16 10.1186/1471-2458-7-16 17266745 PMC1800840

[B50] Eisenmann JC Bartee RT Smith DT Welk GJ Fu Q Combined influence of physical activity and television viewing on the risk of overweight in US youth Int J Obes (Lond) 2008 32 4 613 618 10.1038/sj.ijo.0803800 18209737

[B51] Biddle SJ King J Yates T TV viewing, but not total sedentary behaviour, is associated with adverse cardiometabolic biomarkers in adolescents Evid Based Nurs 2012 Jun 12 Epub ahead of print 10.1136/ebnurs-2012-100613 22691407

[B52] Barabasi AL Network medicine–from obesity to the "diseasome" N Engl J Med 2007 357 4 404 407 10.1056/NEJMe078114 17652657

[B53] Glass TA Population based study of social and productive activities as predictors of survival among elderly Americans (vol 319, pg 478, 1999) Br Med J 1999 319 7217 1130 1130 10.1136/bmj.319.7208.478 PMC28199 10454399

[B54] Hardy R Wills AK Wong A Elks CE Wareham NJ Loos RJ Kuh D Ong KK Life course variations in the associations between FTO and MC4R gene variants and body size Hum Mol Genet 2010 19 3 545 552 10.1093/hmg/ddp504 19880856 PMC2798720

[B55] Mei H Chen W Srinivasan SR Jiang F Schork N Murray S Smith E So JD Berenson GS FTO influences on longitudinal BMI over childhood and adulthood and modulation on relationship between birth weight and longitudinal BMI Hum Genet 2010 128 6 589 596 10.1007/s00439-010-0883-7 20811910

[B56] Lajunen HR Kaprio J Keski-Rahkonen A Rose RJ Pulkkinen L Rissanen A Silventoinen K Genetic and environmental effects on body mass index during adolescence: a prospective study among Finnish twins Int J Obes (Lond) 2009 33 5 559 567 10.1038/ijo.2009.51 19337205 PMC2704063

[B57] Elks CE Loos RJ Hardy R Wills AK Wong A Wareham NJ Kuh D Ong KK Adult obesity susceptibility variants are associated with greater childhood weight gain and a faster tempo of growth: the 1946 British birth cohort study Am J Clin Nutr 2012 95 5 1150 1156 10.3945/ajcn.111.027870 22456663 PMC3325838

[B58] Li S Zhao JH Luan J Ekelund U Luben RN Khaw KT Wareham NJ Loos RJ Physical activity attenuates the genetic predisposition to obesity in 20,000 men and women from EPIC-Norfolk prospective population study PLoS Med 2010 Aug 31 7 8 pii: e1000332 10.1371/journal.pmed.1000332 PMC2930873 20824172

[B59] Janssens AC Moonesinghe R Yang Q Steyerberg EW van Duijn CM Khoury MJ The impact of genotype frequencies on the clinical validity of genomic profiling for predicting common chronic diseases Genetics in medicine: official journal of the American College of Medical Genetics 2007 9 8 528 535 10.1097/GIM.0b013e31812eece0 17700391

[B60] Haberstick BC Lessem JM McQueen MB Boardman JD Hopfer CJ Smolen A Hewitt JK Stable genes and changing environments: body mass index across adolescence and young adulthood Behav Genet 2010 40 4 495 504 10.1007/s10519-009-9327-3 20087641 PMC2989725

[B61] Kilpelainen TO Qi L Brage S Sharp SJ Sonestedt E Demerath E Ahmad T Mora S Kaakinen M Sandholt CH Physical activity attenuates the influence of fto variants on obesity risk: a meta-analysis of 218,166 adults and 19,268 children PLoS Med 2011 8 11 e1001116 10.1371/journal.pmed.1001116 22069379 PMC3206047

[B62] Faith MS Rhea SA Corley RP Hewitt JK Genetic and shared environmental influences on children's 24-h food and beverage intake: sex differences at age 7 y Am J Clin Nutr 2008 87 4 903 911 18400713 10.1093/ajcn/87.4.903

[B63] Bjorntorp P Do stress reactions cause abdominal obesity and comorbidities? Obesity reviews: an official journal of the International Association for the Study of Obesity 2001 2 2 73 86 10.1046/j.1467-789x.2001.00027.x 12119665

[B64] Bowen RS Turner MJ Lightfoot JT Sex hormone effects on physical activity levels: why doesn't Jane run as much as Dick? Sports Med 2011 41 1 73 86 10.2165/11536860-000000000-00000 21142285 PMC3050489

[B65] Stutzmann F Cauchi S Durand E Calvacanti-Proenca C Pigeyre M Hartikainen AL Sovio U Tichet J Marre M Weill J Common genetic variation near MC4R is associated with eating behaviour patterns in European populations Int J Obes (Lond) 2009 33 3 373 378 10.1038/ijo.2008.279 19153581

[B66] Wardle J Carnell S Haworth CM Farooqi IS O'Rahilly S Plomin R Obesity associated genetic variation in FTO is associated with diminished satiety J Clin Endocrinol Metabol 2008 93 9 3640 3643 10.1210/jc.2008-0472 18583465

[B67] Timpson NJ Emmett PM Frayling TM Rogers I Hattersley AT McCarthy MI Smith GD The fat mass- and obesity-associated locus and dietary intake in children Am J Clin Nutr 2008 88 4 971 978 18842783 10.1093/ajcn/88.4.971 PMC4773885

