# Supplementary material for: Leisure time activities in adolescence in the presence of susceptibility genes for obesity: risk or resilience against overweight in adulthood? The HUNT study
Source: BMC Public Health. 2012 Sep 22;12:820. doi: 10.1186/1471-2458-12-820 (PMC3491037; doi:10.1186/1471-2458-12-820)
Supplement: Additional file 1 — Table S1.Genotype information and quality control statistics for the 12 obesity-susceptibility SNP. Table S2 Characteristics of baseline adolescent population who met the inclusion criteria and participated in the follow-up study as adults. Prevalence in numbers or percentages (%), distribution in mean and standard deviation (SD). Table S3 Characteristics of follow-up population who met the inclusion criteria and participated in the baseline study as adolescents. Prevalences are expressed in percentages (%), distribution in mean and standard deviation m (SD). Table S4. Associations between the index for cultural activities at baseline and the z-scores of body mass index (BMI), waist circumference (WC) and waist hip ratio (WHR) eleven years later as adults. Employed GEE linear type model. (N: all 1450 adolescents). Table S5. Associations between the index for social activities at baseline and the z-scores of body mass index (BMI), waist circumference (WC) and waist hip ratio (WHR) eleven years later as adults. Employed GEE linear type model. (N: all 1450 adolescents). Table S6. Associations between the index for cultural activities (without TV-viewing) at baseline and the z-scores of body mass index (BMI), waist circumference (WC), and waist-hip ratio (WHR) eleven years later as adults. Employed GEE linear type model. (N: 1123 normal-weight adolescents). (DOCX 33 kb) [file 1471-2458-12-820-S1.docx]

|  | | | | | | | | | | | | | |  |  |  |  |  |  |  |  |  |  |  |
| --- | --- | --- | --- | --- | --- | --- | --- | --- | --- | --- | --- | --- | --- | --- | --- | --- | --- | --- | --- | --- | --- | --- | --- | --- |
| Table S1. Genotype information and quality control statistics for the 12 obesity-susceptibility SNPs | | | | | | | | | | | |  | | |  | | |  | |  |  |  | |  |
| ___________________________________________________________________________________________________________________________________________________________________________________________________________________________________________________________________________ | | | | | | | | | | | | | | | | | | | | | | | | |
| SNP | | Chrom. | Position | Nearby gene | Total n | Effect allele | Effect allele | Other allele | Article |  | call rate | genotype freq | |  | | |  | HWE | |  |  |  |  |  |
|  | |  |  |  |  |  | frequency |  |  |  |  | Risk allele - homozygous | | heterozygous | | | Other allele homozygous | | |  |  |  |  |  |
|  | |  |  |  |  |  | % |  |  |  |  | % | | % | | | % | p-value | |  |  |  |  |  |
| rs 2815752 | | 1 | [72585028](http://hapmap.ncbi.nlm.nih.gov/cgi-perl/gbrowse/hapmap27_B36/?name=chr1:72585028..72585028) | NEGR1 | 1726 | A | 59.2 | G | 1,6 |  | 99.7 | 34.5 | | 49.3 | | | 16.2 | 0.5259 | |  |  |  |  |  |
| rs 6548238 | | 2 | [624905](http://hapmap.ncbi.nlm.nih.gov/cgi-perl/gbrowse/hapmap27_B36/?name=chr2:624905..624905) | TMEM18 | 1720 | C | 83.7 | T | 1,6 |  | 99.4 | 69.9 | | 27.5 | | | 2.6 | 0.669 | |  |  |  |  |  |
| rs 7566605 | | 2 | [118552495](http://hapmap.ncbi.nlm.nih.gov/cgi-perl/gbrowse/hapmap27_B36/?name=chr2:118552495..118552495) | INSIG2 | 1714 | C | 35.6 | G | 9 |  | 99.0 | 13.6 | | 44.0 | | | 42.4 | 0.1221 | |  |  |  |  |  |
| rs 10195252 | | 2 | [165221337](http://hapmap.ncbi.nlm.nih.gov/cgi-perl/gbrowse/hapmap27_B36/?name=chr2:165221337..165221337) | GRB14 | 1721 | T | 58 | C | 11 |  | 99.4 | 33.6 | | 48.8 | | | 17.7 | 0.9613 | |  |  |  |  |  |
| rs 10938397 | | 4 | [44877284](http://hapmap.ncbi.nlm.nih.gov/cgi-perl/gbrowse/hapmap27_B36/?name=chr4:44877284..44877284) | Near GNPDA2 | 1720 | G | 38.8 | A | 1,6 |  | 99.4 | 15.2 | | 47.3 | | | 37.6 | 0.7656 | |  |  |  |  |  |
| rs 987237 | | 6 | [50911009](http://hapmap.ncbi.nlm.nih.gov/cgi-perl/gbrowse/hapmap27_B36/?name=chr6:50911009..50911009) | Near TFAP2B | 1702 | G | 17.7 | A | 10 |  | 98.4 | 3.3 | | 28.8 | | | 67.9 | 0.7454 | |  |  |  |  |  |
| rs 545854 | | 8 | [9897490](http://hapmap.ncbi.nlm.nih.gov/cgi-perl/gbrowse/hapmap27_B36/?name=chr8:9897490..9897490) | MSRA | 1727 | G | 17.8 | C | 10 |  | 99.8 | 2.9 | | 29.8 | | | 67.3 | 0.4684 | |  |  |  |  |  |
| rs 6265 | | 11 | [27636492](http://hapmap.ncbi.nlm.nih.gov/cgi-perl/gbrowse/hapmap27_B36/?name=chr11:27636492..27636492) | BDNF | 1727 | C | 81.6 | T | 8 |  | 99.8 | 66.5 | | 30.3 | | | 3.2 | 0.9367 | |  |  |  |  |  |
| rs 10838738 | | 11 | [47619625](http://hapmap.ncbi.nlm.nih.gov/cgi-perl/gbrowse/hapmap27_B36/?name=chr11:47619625..47619625) | MTCH2 | 1723 | G | 36.3 | A | 1,6 |  | 99.6 | 13.9 | | 44.7 | | | 41.4 | 0.221 | |  |  |  |  |  |
| rs 1121980 | | 16 | [52366748](http://hapmap.ncbi.nlm.nih.gov/cgi-perl/gbrowse/hapmap27_B36/?name=chr16:52366748..52366748) | FTO | 1726 | A | 44.4 | G | 1,3 |  | 99.7 | 18.9 | | 51.0 | | | 30.1 | 0.1391 | |  |  |  |  |  |
| rs 571312 | | 18 | [55990749](http://hapmap.ncbi.nlm.nih.gov/cgi-perl/gbrowse/hapmap27_B36/?name=chr18:55990749..55990749) | MC4R | 1715 | A | 26.9 | C | 5 |  | 99.1 | 6.6 | | 40.6 | | | 52.8 | 0.1315 | |  |  |  |  |  |
| rs 11084753 | | 19 | [39013977](http://hapmap.ncbi.nlm.nih.gov/cgi-perl/gbrowse/hapmap27_B36/?name=chr19:39013977..39013977) | Near KCTD15 | 1716 | G | 69.5 | A | 1,6 |  | 99.2 | 47.6 | | 43.7 | | | 8.7 | 0.1818 | |  |  |  |  |  |
| Chrom: Chromosome; Article reference: 1) Loos et al., 2009; 2) Hakanen et al., 2009 3) Hinney et al., 2007; 4) Loos et al., 2008; 5) Grant et al., 2009; 6) Willer et al., 2009; 7) Zhao et al., 2009;  8) Thorleifson et al., 2009; 9) Andreassen et al., 2008; 10) Lindgren et al., 2009; 11) Heid et al., 2010; HWE: Hardy-Weinberg equilibrium; Call-rate: rate of successful genotyping. | | | | | | | | | | | | | | | | | | | | | | |  |  |

| Table S2. | | Characteristics of baseline adolescent population who met the inclusion criteria and participated in the follow-up study as adults. Prevalence in numbers or percentages (%), distribution in mean and standard deviation (SD). | | | | | | | | | |
| --- | --- | --- | --- | --- | --- | --- | --- | --- | --- | --- | --- |
|  |  | | | | | | | | | |  |
|  | |  | |  | | |  | Girls (603) | Boys ( 520) |  | |
|  | |  | |  | | |  |  |  |  | |
|  | |  | |  | | |  |  |  |  | |
| Age | |  | |  | | |  | 16.0 (1.8) | 15.9 (1.8) |  | |
| Social activities | |  | |  | | |  | 10.6(2.3) | 10.5 (2.5) |  | |
| Lowest participation | | | | | | | 1 | 17 % | 22 % |  | |
|  | |  | |  | | | 2 | 29 % | 26 % |  | |
|  | |  | |  | | | 3 | 17 % | 13 % |  | |
| Highest participation | | | | | | | 4 | 37 % | 39 % |  | |
|  | | |  |  | | |  |  |  |  | |
| Cultural activities | | |  |  | | |  | 11.3 (1.8) | 10.2 (1.9) |  | |
| Lowest participation | | | | | | | 1 | 8 % | 16 % |  | |
|  | |  | |  | | | 2 | 24 % | 43 % |  | |
|  | |  | |  | | | 3 | 20 % | 19 % |  | |
| Highest participation | | | | | | | 4 | 48 % | 23 % |  | |
|  | | | | |  |  |  |  |  |  | |
| Pubertal Development Status | | | | |  |  |  | 3.4 (0.6) | 3.0 (0.7) |  | |
|  | | | | |  |  |  |  |  |  | |
| Physical activity | | | | | |  | 1 | 71 % | 72 % |  | |
|  | | | |  | |  |  |  |  |  | |
|  | | | |  | |  |  |  |  |  | |
| BMI | | | |  | |  |  | 20.7 (1.9) | 20.4 (1.8) |  | |
| Waist circumference | | | |  | |  |  | 68.8 (5.4) | 73.6 (5.6) |  | |
| Waist-hip ratio | | | |  | |  |  | 0.744 (0.044) | 0.795 (0.045) | | |

___________________________________________________________________________

Physical activity: 1= high level, at least 2 to 3 times a week of doing exercises or sports at an intensity of out of breath or into sweat. 0= low level, less than 2 times a week of doing exercises or sports at an intensity of out of breath or into sweat.

| Social activities: visiting some one you know, receiving a visitor, being out for more than two  hours with friends, being at a meeting or training in an organization or a club. | |
| --- | --- |
| Cultural activities: reading a book you liked, listening to music or playing an instrument  longer than 15 minutes, watching TV or a video, and doing homework or school tasks longer than an hour. |  |

| Table S3. | Characteristics of follow-up population who met the inclusion criteria and participated in the baseline study as adolescents. Prevalences are expressed in percentages (%), distribution in mean and standard deviation m (SD). |
| --- | --- |
| _______________________________________________________________________________ | |

|  |  |  | Females | Males |  |
| --- | --- | --- | --- | --- | --- |
|  |  |  |  |  |  |
| BMI | 1 |  | 36 % | 50 % |  |
|  |  |  |  |  |  |
| WC | 1 |  | 60 % | 31 % |  |
|  |  |  |  |  |  |
| WHR | 1 |  | 64 % | 33 % |  |
|  |  |  |  |  |  |
|  |  |  |  |  |  |
| Physical activity | 1 |  | 61 % | 60 % |  |
|  |  |  |  |  |  |
| Age |  |  | 27.2 (1.9) | 27.2 (1.9) |  |
|  |  |  |  |  |  |
| BMI |  |  | 24.4 (3.7) | 25.3 (3.2) |  |
| WC |  |  | 83.6 (11.5) | 89.8 (9.0) |  |
| WHR |  |  | 0.827 (0.074) | 0.876 (0.054) | |

BMI ≥ 25 kg/m^2^, Waist circumference (WC) > 80cm for females and WC > 94 cm for men, Waist-hip ratio (WHR) over 0.80 in women and over 0.90 in men was defined as overweight (1).

Physical activity: 1= high level, at least 2 to 3 times a week of doing exercises or sports at an intensity of out of breath or into sweat.

Table S4. Associations between the index for cultural activities at baseline and the z-scores of body mass index (BMI), waist circumference (WC) and waist hip ratio (WHR) eleven years later as adults. Employed GEE linear type model. (N: all 1450 adolescents)

|  |  |  |  | Girls |  |  |  | Boys |  |  |
| --- | --- | --- | --- | --- | --- | --- | --- | --- | --- | --- |
|  |  | B | SE | P | CI (95%) | B | SE | P | CI (95%) |  |
| BMI | Model 2 | -.027 | .023 | .240 | -.07 to .020 | -.005 | .029 | .870 | -.06 to .05 |  |
|  |  |  |  |  |  |  |  |  |  |  |
| WC | Model 2 | -.039 | .022 | .070 | -.08 to .003 | -.005 | .027 | .840 | -.06 to .05 |  |
|  | Model 3 | -.035 | .020 | .080 | -.07 to .005 | .003 | .026 | .910 | -.05 to .05 |  |
| WHR | Model 2 | -.041 | .022 | .060 | -.09 to .002 | -.012 | .026 | .660 | -.06 to .04 |  |
|  | Model 3 | -.047 | .018 | .010 | -.08 to -.01 | .008 | .022 | .720 | -.04 to .05 |  |
|  |  |  |  |  |  |  |  |  |  |  |

Girls n=775; Boys n=675. Excluded: pregnant at follow-up and disabled physically or psychologically in daily activities.

Model 2: Adjusted for pubertal development at baseline, physical activity change from adolescence to adulthood, and socio-economic status at follow-up.

Model 3: Adjusted additionally for baseline BMI- value. In case of WC respectively WHR z-scores in young adulthood adjusted for BMI z-scores in adolescence. In the GEE model with BMI as outcome it is statistically not recommendable to introduce baseline BMI, because this variable is already included.

Table S5. Associations between the index for social activities at baseline and the z-scores of body mass index (BMI), waist circumference (WC) and waist hip ratio (WHR) eleven years later as adults. Employed GEE linear type model. (N: all 1450 adolescents)

|  |  |  |  | Girls |  |  |  | Boys |  |  |
| --- | --- | --- | --- | --- | --- | --- | --- | --- | --- | --- |
|  |  | B | SE | P | CI (95%) | B | SE | P | CI (95%) |  |
| BMI | Model 2 | -.006 | .020 | .770 | -.040 to .03 | .037 | .015 | .010 | .008 to .070 |  |
|  |  |  |  |  |  |  |  |  |  |  |
| WC | Model 2 | .014 | .019 | .470 | -.020 to .05 | .035 | .015 | .020 | .006 to .070 |  |
|  | Model 3 | .013 | .008 | .120 | -.003 to .03 | .015 | .007 | .020 | .002 to .030 |  |
| WHR | Model 2 | .017 | .019 | .380 | -.020 to .05 | .030 | .017 | .070 | -.002 to .060 |  |
|  | Model 3 | .012 | .014 | .380 | -.020 to .04 | .034 | .013 | .020 | .006 to .090 |  |
|  |  |  |  |  |  |  |  |  |  |  |

Girls n=775; Boys n=675. Excluded: pregnant at follow-up and disabled physically or psychologically in daily activities.

Model 2: Adjusted for pubertal development at baseline, physical activity change from adolescence to adulthood, and socio-economic status at follow-up.

Model 3: Adjusted additionally for baseline BMI- value. In case of WC respectively WHR z-scores in young adulthood adjusted for BMI z-scores in adolescence. In the GEE model with BMI as outcome it is statistically not recommendable to introduce baseline BMI, because this variable is already included.

| Table S6. Associations between the index for cultural activities (without TV-viewing) at baseline and the z-scores of body mass index (BMI), | | | | | | | | | | | | | | |  |
| --- | --- | --- | --- | --- | --- | --- | --- | --- | --- | --- | --- | --- | --- | --- | --- |
| waist circumference (WC), and waist-hip ratio (WHR) eleven years later as adults. Employed GEE linear type model. (N: 1123 normal-weight adolescents) | | | | | | | | | | | | | | | |
| ________________________________________________________________________________________________ | | | | | | | | | | | | | | | |
|  |  |  |  | Girls |  |  |  | Boys |  | |  |  |  |  |  |
|  |  |  |  |  |  |  |  |  |  | |  |  |  |  |  |
|  |  | B | SE | P | CI (95%) | B | SE | P | CI (95%) | |  |  |  |  |  |
|  |  |  |  |  |  |  |  |  |  | |  |  |  |  |  |
| BMI |  |  |  |  |  |  |  |  |  | |  |  |  |  |  |
| Model 1 |  | -.010 | .014 | .51 | -.04 to .02 | -.005 | .014 | .71 | -.03 to .02 | |  |  |  |  |  |
| Model 2 |  | -.003 | .016 | .86 | -.03 to .03 | .05 | .016 | .73 | -.03 to .04 | |  |  |  |  |  |
|  |  |  |  |  |  |  |  |  |  | |  |  |  |  |  |
|  |  |  |  |  |  |  |  |  |  | |  |  |  |  |  |
| WC |  |  |  |  |  |  |  |  |  | |  |  |  |  |  |
| Model 1 |  | -.029 | .015 | .05 | -.06 to 0 | -.005 | .015 | .73 | -.04 to .02 | |  |  |  |  |  |
| Model 2 |  | -.034 | .018 | .05 | -.07 to 0 | .014 | .017 | .39 | -.02 to .05 | |  |  |  |  |  |
|  |  |  |  |  |  |  |  |  |  | |  |  |  |  |  |
|  |  |  |  |  |  |  |  |  |  | |  |  |  |  |  |
| WHR |  |  |  |  |  |  |  |  |  | |  |  |  |  |  |
| Model 1 |  | -.051 | .017 | .002 | -.08 to -.02 | -.010 | .018 | .58 | -.05 to.03 | |  |  |  |  |  |
| Model 2 |  | -.046 | .020 | .02 | -.08 to -.01 | .027 | .020 | .19 | -.01 to .07 | |  |  |  |  |  |
|  |  |  |  |  |  |  |  |  |  | |  |  |  |  |  |
| Girls n=603; Boys n=520. Excluded: overweight and underweight, pregnant, disabled physically or psychologically in daily activities. | | | | | | | | | | | | |  |  |  |
| Model 1 Unadjusted effect of exposure on the outcome measure at follow-up | | | | | | | | | | | |  |  |  |  |
| Model 2. Adjusted pubertal development at baseline, physical activity change from adolescence to adulthood, and socio-economic status at follow-up. | | | | | | | | | | | | | |  |  |
| Cultural activities: reading a book you liked, listening to music or played an instrument longer than 15 minutes, and doing homework or school tasks longer than an hour. We assigned values from 1 to 4 for each answer-category regarding frequency (Not once (1), Once (2), 2-3 times (3), 4 times or more (4) in the last 7 days) and made a continuous variable by adding the scores for each activity (range 4- 16). | | | | | | | | | |  |  |  |  |  |  |
